# The Psychological and Medical Landscape of Anorexia Nervosa over a Decade: A Retrospective Study of Possible Physical and Psychological Shifts

**DOI:** 10.3390/jcm12237294

**Published:** 2023-11-24

**Authors:** Paolo Meneguzzo, Lorenza Di Pascoli, Maria Cristina Bindolo, Enrico Collantoni, Valentina Meregalli, Tatiana Zanetti, Angela Veronese, Elena Tenconi, Angela Favaro

**Affiliations:** 1 Department of Neuroscience, University of Padova, 35122 Padova, Italy; enrico.collantoni@unipd.it (E.C.); valentina.meregalli@phd.unipd.it (V.M.); elena.tenconi@unipd.it (E.T.); angela.favaro@unipd.it (A.F.); 2 Padova Neuroscience Center, University of Padova, 35122 Padova, Italy; 3 Unit of Internal Medicine and Hepatology (UIMH), Department of Medicine, Padova University Hospital, 35128 Padova, Italy; 4 Eating Disorders Unit, Padova University Hospital, 35128 Padova, Italy

**Keywords:** anorexia nervosa, COVID-19, bulimia, obsessive–compulsive, interoceptive awareness, hospitalization, adolescent

## Abstract

In recent years, the definition, clinical characteristics, and psychological aspects of anorexia nervosa (AN) have undergone notable changes, influenced by various factors such as biology, psychology, and the environment. The COVID-19 pandemic is one such external factor that has been preliminarily identified as affecting the clinical presentation of AN. This study specifically aims to assess the alterations in psychological and medical features observed in individuals with AN during the pandemic. This study is a retrospective case review of 252 people diagnosed with AN from two different cohorts: 2010–2012 and 2021. A comparison of psychological and medical data was conducted to identify specific differences in the initial clinical evaluation. Additionally, different effects of the pandemic on age cohorts were assessed, with a focus on distinctions between adolescents and adults. Results revealed that the pandemic cohort had a higher number of new cases, with patients being younger and experiencing more severe psychological symptoms. Hospitalization rates upon first access were also elevated, but no significant differences in medical values were observed. Adolescents during the pandemic showed increased episodes of binge eating, decreased obsessive–compulsive symptoms, and greater difficulty in interoceptive awareness. This study reveals distinct differences in symptomatology among patients, with a particular focus on psychological characteristics such as impulsive behaviors and interoceptive awareness. These behavioral and interoceptive changes could have potential considerations in the treatment pathway. Further investigations are warranted to gain a deeper understanding of the evolving clinical presentation of anorexia nervosa after the pandemic.

## 1. Introduction

In recent decades, numerous authors have explored the noteworthy transformations observed in the clinical presentation of various mental illnesses. The continual evolution of the clinical, definitional, and psychological aspects of anorexia nervosa and schizophrenia over recent decades, particularly within conditions such as schizophrenia, mood disorders, and anorexia nervosa (AN), is vital, as it enhances diagnostic precision, informs treatment advancements, reduces stigma, fosters research and innovation, promotes public health awareness, guides policy development, and ensures the ongoing education of mental health professionals [1]. The concept of change in the context of AN has a longstanding historical backdrop [1] characterized by substantial contributions from prominent figures such as Hilde Bruch (1970), who incorporated psychological factors related to distressing life experiences, and Gerald Russell (1985), who elucidated the central role of concerns regarding body weight and the fear of becoming overweight. More recently, a growing body of research has highlighted the etiological role of genes [2] to the extent that the term “metabo-psychiatric disorder” has been proposed. However, a purely biological approach appears insufficient to comprehensively explain AN given the crucial role played by the interplay between biological, psychological, and sociocultural elements. This interaction is exemplified by phenomena such as the decrease in the age of onset [3] and potential ecophenotype effects resulting from life events, such as trauma [4]. Traumatic events can have both acute and long-term effects, which have been documented in the field of eating disorders (EDs) in the last few years during the COVID-19 pandemic [5,6].

Indeed, people with EDs have been identified as one of the populations significantly affected by the COVID-19 pandemic, including individuals with a history of EDs and those susceptible to developing EDs [7,8]. The COVID-19 pandemic has caused significant disruptions in people’s lives worldwide, with adolescents’ mental health being particularly impacted [9]. The implementation of widespread restrictions on public and private life to contain the spread of the pandemic has profoundly affected mental well-being among the general population and, more severely, individuals with a history of mental disorders [10]. The pandemic’s effects on EDs can be attributed to the direct psychological consequences of confinement measures and the challenges faced by ED services in adapting during the pandemic. Indeed, ED services worldwide have reported being overwhelmed, with an upsurge in requests for support and treatment [11]. Individuals with EDs have reported increased distress due to their vulnerability to stressful events, more severe internalizing symptoms, and disruptions in therapeutic relationships [12]. The pandemic has been described as a traumatic event, especially for individuals with EDs, who present lasting effects even one year after the initial lockdown measures [6,13]. Numerous studies have reported increased hospitalizations related to EDs and worsened ED symptoms during the pandemic [14,15]. Specific factors contributing to these effects include reduced access to care and treatment, changes in routine and loss of structure, the negative influence of the media, and social isolation [16,17]. The existing literature suggests age and timing specificity regarding the effects of the pandemic, such as potential improvements post-lockdown, but further research is needed, particularly focusing on the severity of presentation [16,18].

Looking specifically at AN, there is a growing body of evidence reporting a striking increase in the occurrence of new cases among young individuals during the pandemic, coinciding with the timing of social restrictions [19]. Several authors have already documented a change in the psychological presentation of AN during the pandemic [15,20], highlighting specific psychological burdens marked by heightened somatization, increased interpersonal sensitivity, greater levels of depression, different functioning profiles (i.e., personality traits), and heightened body uneasiness. However, it is crucial to note that AN is also marked by significant physical symptoms, encompassing cardiovascular, ionic, and hormonal aspects [21]. These symptoms have become challenging to assess due to the social distancing measures and service closures brought about by the pandemic [22]. Preliminary evidence suggests the absence of more severe medical comorbidities despite the presence of an increased number of hospitalizations [23,24], but studies are limited. Nonetheless, it is important to acknowledge that research in this area remains relatively underexplored, with available studies primarily focusing on the pediatric population and psychological effects, neglecting medical features [25].

Therefore, the aim of this study is to compare the medical and psychological records of individuals evaluated for AN during the first year of the COVID-19 pandemic with a historical cohort, looking at differences related to age. The objective is to identify specific patterns that might characterize AN patients at different time points because our hypothesis suggests a change in clinical presentation after the COVID-19 pandemic, with a more severe medical and psychological presentation, justifying the increased number of hospitalizations and treatment requests.

## 2. Materials and Methods

### 2.1. Study Design

We conducted a retrospective analysis of clinical records from patients admitted for AN at the University Hospital of Padova (Italy). We included data from clinical records of all patients who were visited for the first time during two specific time periods: January 2010 to December 2012, and January to December 2021. The study included patients who were consecutively referred to the Eating Disorders Unit of the Padova University Hospital, which provides outpatient care to a catchment area of over 200,000 people in Padova and the surrounding province. The Unit has connections with the inpatient services of Medical Clinic 5 and the neuropsychiatric inpatient service of the University Hospital of Padova, where patients can receive hospitalization for acute conditions. Considering the increase in requests, we included a two-year time frame for the older cohort to ensure comparable sample sizes between the two cohorts.

The inclusion criteria for this study were as follows: (a) meeting the full diagnostic criteria for AN as assessed using the structured clinical interview for DSM-5; (b) being at least 14 years old and providing informed consent (or obtaining consent from legal guardians if under 18 years of age) for the use of clinical data at the time of initial evaluation; (c) not receiving treatment for an eating disorder in the two years preceding admission. Trained psychiatrists conducted all diagnoses using the structured clinical interview for DSM-5 (SCID-5) for the 2021 cohort. For the 2010–2012 cohort, structured clinical interviews based on the DSM-IV-TR criteria for AN or eating disorders not otherwise specified (EDNOS) were reassessed using the DSM-5 criteria to determine their inclusion or exclusion from this study.

All data were analyzed anonymously. Written informed consent was obtained from all individual participants included in this study (or their guardians if underage) for the use of their clinical data for research purposes. The study was carried out in accordance with the Declaration of Helsinki and its subsequent amendments, and the Padova Ethics Committee (327n/AO/23) approved the study design.

### 2.2. Data

Medical records were examined for the following variables: age, sex, weight, height, age of onset, duration of the disorder, presence of purging behaviors (diuretics and vomiting), type of admission (inpatient or outpatient), and resting heart rate (the average of three evaluations). All data were recorded during the first medical evaluation of the patients. Considering the severe effects that AN has on different systems and human body apparatuses, we collected different clinical data. The results were reviewed, including leukocyte, lymphocyte, erythrocyte and platelet counts; hemoglobin, fasting blood glucose, creatinine, blood urea nitrogen, sodium, potassium, chloride, magnesium, calcium, phosphorus, alanine aminotransferase (ALT), aspartate aminotransferase (AST), gamma-glutamyl transpeptidase (GGT); total bilirubin, iron, ferritin, zinc, and thyroid stimulating hormone (TSH); and free thyroxine (fT4) and free triiodothyronine (fT3).

Psychological evaluations of participants were conducted through clinical interviews and using different self-report questionnaires routinely used in the initial assessment of patients. This included the Eating Disorder Inventory (EDI), a 64-item self-report questionnaire used to assess the presence of eating disorder psychopathology [26]. The EDI consists of eight subscales: drive to thinness, bulimia, body dissatisfaction, ineffectiveness, perfectionism, interpersonal distrust, interoceptive awareness, and maturity fears. Higher scores represent higher levels of psychopathology. To evaluate general psychological health status, we used the revised Symptom Checklist (SCL-90-R), a 90-item self-report questionnaire that includes nine subscales: somatization, obsessive–compulsive, interpersonal sensitivity, depression, anxiety, hostility, phobic anxiety, psychoticism, and sleep difficulties [27]. Finally, the Tridimensional Personality Questionnaire (TPQ), a self-reported questionnaire, was used to assess specific personality traits. It consists of three subscales: novelty seeking, harm avoidance, and reward dependence [28].

### 2.3. Statistical Plan

Data were reported as means and standard deviations or percentages, as appropriate. Effect sizes for significant differences were evaluated using Cohen’s *d* and Cramer’s *V* for the chi-square test. A second level of analysis involved evaluating the interaction between variables and age using a MANCOVA approach, with an age cut-off of 18 years to distinguish adolescence. Logistic regression was employed, using the variables that demonstrated significance in multiple comparisons across the entire sample, including both adolescents and adults. The aim was to identify a more concise model elucidating the distinctions between the pre-pandemic and pandemic groups. A significance level of α = 0.05 was assumed for all analyses. Data were analyzed using IBM SPSS Statistics 25.0 software (SPSS, Chicago, IL, USA).

## 3. Results

A total sample of 252 people was included in the present study: 135 from the 2010–2012 cohort (T1) and 117 from the 2021 cohort (T2). No differences were found in the gender or AN subtype distribution. The T2 cohort presented a lower age and a higher rate of patients diagnosed for the first time after hospitalization, but no difference in BMI at our first evaluation. The cohort T2 also showed a lower age of onset and a shorter duration of the disorder. Considering age, we found that 54 individuals in T1 (40.0%) and 68 individuals in T2 (58.1%) were under 18 years old (χ^2^ (1) = 8.240, *p* = 0.004, V = 0.181). See Table 1 for details.

Specific differences were found more in the psychological evaluation than in the medical evaluation; see Table 2 and Table 3 for details.

More severe scores were found in adolescents compared to adults in some psychological variables: interoceptive awareness (F(1,160) = 4.750, *p* = 0.031), obsessive–compulsive (F(1,160) = 5.451, *p* = 0.021), depression (F(1,160) = 4.110, *p* = 0.044), and avoidance of harm (F(1,160) = 4.142, *p* = 0.039). In contrast, the interaction between age and scores was not significant for all other medical and psychological characteristics considered.

### Logistic Regression

This study included three different regressions to examine the factors that can explain the differences between the cohorts. While the first analysis was conducted on the entire group, the sample was then divided according to age, into adolescents and adults, and separate regressions were performed for each group. The results produced only one specific model that could potentially explain the variations observed between the adolescent cohorts, while no specific predictor was found for the adult cohorts or the entire sample.

For the adolescent group, the results showed that the pandemic cohort had a higher probability of presenting with more binge-eating episodes followed by compensatory behaviors, greater difficulties with interoceptive awareness, and lower obsessive–compulsive symptoms. No specific psychological construct was found to be a valid predictor for the entire sample or for the adult group. For more detailed information on these findings, please refer to Table 4.

## 4. Discussion

The primary hypothesis of our study was that patients with AN would exhibit a more severe clinical presentation following the onset of the COVID-19 pandemic. This hypothesis was only partially supported, showing changes in the psychological aspects and stability in the medical parameters. Indeed, we observed a more severe psychological presentation, characterized by increased severity of psychopathological states, in the more recent cohort, with only minor significant differences in medical values. We also noted an increase in treatment requests, more frequent hospitalizations as the initial point of contact, shorter durations of illness, and a younger age. However, despite these factors, no specific clinical parameter demonstrated significantly greater compromise.

During the pandemic, AN symptomatology appeared to exhibit changes in psychological characteristics, but not in medical comorbidities, despite the significant increase in requests for inpatient treatment recorded worldwide. The lack of substantial clinical differences in medical variables is consistent with findings from other studies on pediatric patients with AN [23] and underscores the significant role of psychological factors in the clinical severity of AN. Furthermore, this highlights, once again, the importance of simultaneously considering both medical and psychological aspects throughout all stages of AN treatment.

Numerous reviews of the literature have suggested an increase in the prevalence and/or severity of ED symptoms during the COVID-19 period [29]. According to our data, this deterioration appears to be more closely related to psychological functioning than physical aspects, with no specific changes in the medical profile. However, the decrease in the duration of the disorder before the initial examination could indicate the presence of more acute changes in eating behaviors, such as fasting or fasting-accelerated dieting [30]. Furthermore, fasting usually results in rapid weight suppression, which can explain the increase in hospitalization, as well as psychopathological severity [31]. Other studies in the field of EDs have reported similar findings [14,15], indicating the possible existence of specific changes in psychopathological presentation during the acute phase of the pandemic that could be due to the presence of specific vulnerabilities or psychological effects. Another possible explanation for the reduction in the duration of the disorder is related to the social restrictions that brought families together in confined spaces. While this could potentially contribute to the manifestation of symptoms, it may have also made these symptoms more noticeable to parents earlier [32].

When examining specific psychopathological changes, we observed that only in the adolescent subgroup were we able to find specific predictors of changes. Adolescents with AN after the pandemic had higher impulsivity, less obsessive–compulsive symptoms, and more difficulty with their body awareness. It appears that psychological functioning was more characterized by behavioral and bodily aspects than mental functioning [33]. Looking at the effects of the COVID-19 pandemic, similar studies have also reported an association between body-related symptoms and the pandemic [14], as well as a higher prevalence of binge-eating episodes [20] and a perceived “loss of control” [34]. From this perspective, the COVID-19 pandemic may have had a significant impact on the clinical presentation of AN as a result of sudden confinements, disruption of daily routines, and changes in interpersonal relationships and family dynamics [35,36]. These changes should be taken into account in future longitudinal studies aimed at assessing the evolving psychological aspects of the clinical presentation of AN. Personalized treatments are the future perspective in the ED field [37]. If substantiated, the presence of a more impulsive symptomatology, coupled with a decrease in interoceptive abilities, should be taken into account in future studies and considered in the context of clinical practice. In this regard, cognitive behavioral therapies have already incorporated third-wave strategies that appear to be effective for these clinical profiles, although further studies are necessary to establish their efficacy conclusively [38].

The role of psychological factors in the increase in AN requests, as indicated by our data, corresponds to findings from multiple studies conducted in different regions globally [15,23,39]. These findings reinforce the notion that ED services should incorporate a comprehensive approach that integrates both psychological and medical aspects. Additionally, it is crucial for ED services to possess the ability to swiftly adapt and respond to shifts in clinical presentation, especially in the face of potential future disruptive events that could disrupt life trajectories, requiring quick adaptation like online services or changes in treatment options [40]. This adaptability is essential to prevent the overwhelming challenges experienced during the current situation, with people reporting feelings of abandonment from ED services [8].

### 4.1. Limitations

It is important to acknowledge the limitations of our study. The findings may not be generalizable beyond the specific study period, and the retrospective design prevented us from establishing causal relationships between COVID-19 and the onset or exacerbation of AN. We included two different cohorts of patients, with a long period of time between them. While this might be considered a limitation, it also provided us with the opportunity to evaluate changing trends in clinical presentation. Finally, a specific limitation is associated with the potential changes in the instruments used in the laboratory for assessing biological features. Despite these limitations, our findings contribute to the growing body of evidence linking the COVID-19 pandemic, hospital utilization patterns, and illness symptoms in young people.

### 4.2. Future Directions

Although this retrospective analysis does not allow us to isolate the impact of COVID-19 from other potentially significant concurrent factors, these findings contribute to our understanding of how the pandemic could have influenced individuals who needed specialized treatment for AN. Future research is crucial to gain a deeper understanding of the factors that contribute to the increased demand for specialized AN treatment and to understand the stability of the results we found. To fully capture the patterns and rates of AN onset after the pandemic, as well as long-standing trends, it is important to conduct multimethod studies. These studies should incorporate various research approaches and methodologies to examine the complex biopsychosocial factors that play a role in the onset of the disorder. Additionally, exploring multiple stakeholder perspectives, such as patients, caregivers, healthcare professionals, and experts, can provide valuable information on the underlying mechanisms and inform targeted interventions and prevention strategies.

A specific aspect that future studies should incorporate is the evaluation of the differences in symptomatology between patients from rural and urban areas, including those related to the pandemic crisis. This might help to understand changes and clinical features.

## 5. Conclusions

Taken together, this study suggests that during the COVID-19 pandemic, young patients with AN exhibited changes in clinical presentation characterized by a lower age of onset, shorter duration of the disorder, more episodes of binge eating, and difficulties related to interoception, while obsessive–compulsive symptoms appeared to decrease. No changes were recorded in the medical presentation or in adult patients. Continuously monitoring the long-term and potentially delayed effects of the pandemic on the development and progression of AN, as well as trends in hospitalization among young individuals, is crucial for identifying the stability of these changes. Moreover, future studies could help to develop appropriate support and interventions in response to future COVID-19 outbreaks or other global crises that may hinder access to care.

## Figures and Tables

**Table 1:** Demographic characteristics of the sample.

	T1 (*n* = 135)	T2 (*n* = 117)	*t*	*p* (*d*)
Age, years	22.40 (9.69)	20.18 (7.20)	2.031	0.043 (0.260)
BMI, kg/m^2^	16.34 (1.89)	16.56 (2.41)	−0.792	0.429
Gender, F	129 (95.6%)	112 (95.7%)	0.918 *	0.638
AN restrictive subtype	104 (77.0%)	91 (77.7%)	1.518 *	0.218
Hospitalization as first contact	9 (6.6%)	36 (30.8%)	24.824	<0.001 (0.314 §)
Age of onset, years	18.55 (6.14)	16.85 (5.04)	2.246	0.026 (0.303)
Duration of the disorder, months	24.45 (45.33)	14.59 (20.38)	2.065	0.041 (0.280)

Table reports means and standard deviations. *: χ^2^, §: Cramer’s *V*.

**Table 2:** Medical characteristics of the sample.

	T1 (*n* = 135)	T2 (*n* = 117)	*t*	*p*
Heart rate (bpm)	60.92 (14.25)	61.15 (12.23)	−0.119	0.906
Leukocytes (×10^9^/L)	5.35 (2.22)	4.94 (1.66)	1.409	0.160
Erythrocytes (×10^9^/L)	4.39 (0.60)	4.51 (0.54)	−1.472	0.143
Hemoglobin (g/dL)	13.37 (6.29)	13.50 (1.58)	−0.193	0.847
Hematocrit (%)	39.13 (3.74)	40.17 (3.37)	−1.474	0.131
Platelets (×10^12^/L)	252.84 (237.60)	232.22 (58.00)	0.808	0.420
Creatinine (umol/L)	72.88 (13.59)	69.72 (12.78)	1.550	0.123
Sodium (mmol/L)	140.09 (5.23)	139.22 (3.20)	1.286	0.200
Potassium (mmol/L)	4.12 (0.51)	4.12 (0.51)	0.003	0.998
Chlorine (mmol/L)	102.39 (4.67)	102.36 (3.07)	0.043	0.966
Glycemia (mmol/L)	4.17 (0.57)	4.43 (0.76)	−1.251	0.096
AST (U/L)	29.25 (16.81)	25.58 (14.14)	1.516	0.131
ALT (U/L)	31.99 (32.98)	25.32 (13.61)	1.686	0.094
GGT (U/L)	18.24 (15.05)	13.74 (9.20)	1.925	0.057
Total bilirubin (umol/L)	17.95 (22.28)	22.05 (28.19)	−0.650	0.518
TSH (mUI/L)	1.77 (1.17)	1.91 (0.95)	−0.754	0.452
fT3 (pmol/L)	2.99 (1.12)	3.51 (3.53)	−0.745	0.459
fT4 (pmol/L)	13.30 (5.17)	12.67 (3.46)	0.704	0.483

Bpm: beats per minute; AST: aspartate transaminase; ALT: alanine aminotransferase; GGT: gamma-glutamyl transferase; TSH: thyroid stimulating hormone; fT3: free triiodothyronine; fT4: free thyroxine. The table reports means with standard deviations in brackets, and Cohen’s *d* is reported only for significant differences between cohorts.

**Table 3:** Psychological characteristics of the sample.

	T1	T2	*t*	*p* (*d*)
EDI				
Drive to thinness	9.38 (7.40)	13.49 (6.61)	−3.884	<0.001 (0.586)
Bulimia	2.14 (3.33)	3.49 (3.37)	−2.668	<0.001 (0.403)
Body dissatisfaction	10.12 (7.69)	15.13 (7.60)	−4.351	<0.001 (0.655)
Ineffectiveness	7.05 (6.33)	11.66 (7.66)	−4.349	<0.001 (0.656)
Perfectionism	4.49 (3.99)	4.99 (3.65)	−0.857	0.393
Interpersonal distrust	6.46 (4.43)	7.81 (4.35)	−2.047	0.042 (0.307)
Interoceptive awareness	7.31 (6.79)	11.77 (7.53)	−4.141	<0.001 (0.622)
Maturity fears	7.29 (3.99)	8.91 (5.03)	−2.376	0.019 (0.357)
SCL90R				
Somatization	1.07 (0.93)	1.57 (0.89)	−3.637	<0.001 (0.594)
Obsessive–Compulsive	1.35 (0.90)	1.90 (0.92)	−3.963	<0.001 (0.604)
Interpersonal sensitivity	1.30 (0.82)	1.87 (0.88)	−4.453	<0.001 (0.670)
Depression	1.45 (0.91)	2.11 (0.94)	−4.741	<0.001 (0.713)
Anxiety	1.18 (0.88)	1.74 (0.91)	−4.166	<0.001 (0.625)
Hostility	0.89 (0.70)	1.17 (0.78)	−2.504	0.013 (0.378)
Phobic anxiety	0.44 (0.50)	0.86 (0.85)	−3.908	<0.001 (0.602)
Psychoticism	0.81 (0.66)	1.20 (0.69)	−3.888	<0.001 (0.578)
Sleep difficulties	1.33 (1.25)	2.01 (1.25)	−3.556	<0.001 (0.544)
Total score	1.13 (0.69)	1.64 (0.73)	−4.741	<0.001 (0.718)
TPQ				
Novelty seeking	14.97 (5.40)	13.71 (5.06)	1.561	0.120
Harm avoidance	19.95 (6.63)	23.30 (6.60)	−3.284	0.001 (0.506)
Reward dependence	11.72 (3.68)	12.04 (3.64)	−0.555	0.580

EDI: Eating Disorder Inventory; SCL90R: Symptom Checklist; TPQ: Tridimensional Personality Questionnaire. The table reports means with standard deviations in brackets, and Cohen’s *d* was reported only for significant differences between cohorts.

**Table 4:** Multivariate logistic regression analysis.

	B (SE)	OR	95% CI
All samples (R^2^ = 0.170)			
Drive to thinness	0.017 (0.040)	1.017	0.940–1.099
Bulimia	0.001 (0.065)	1.000	0.881–1.135
Body dissatisfaction	0.006 (0.037)	0.936	0.936–1.081
Ineffectiveness	−0.001 (0.042)	0.999	0.919–1.085
Interpersonal distrust	−0.068 (0.053)	0.934	0.841–1.037
Interoceptive awareness	0.042 (0.040)	1.043	0.964–1.128
Maturity fears	0.038 (0.042)	1.039	0.957–1.127
Somatization	0.021 (0.342)	1.021	0.523–1.995
Obsessive–compulsive	−0.321 (0.451)	1.779	0.632–5.010
Interpersonal sensitivity	0.284 (0.383)	1.329	0.628–2.812
Depression	0.576 (0.528)	1.779	0.632–5.010
Anxiety	0.026 (0.427)	1.026	0.444–2.370
Hostility	−0.261 (0.340)	0.770	0.396–1.500
Phobic anxiety	0.397 (0.421)	1.488	0.652–3.396
Psychoticism	−0.294 (0.492)	0.745	0.284–1.956
Sleep difficulties	0.178 (0.180)	1.195	0.839–1.701
Harm avoidance	0.009 (0.034)	1.009	0.945–1.078
Adolescents (R^2^ = 0.627)			
Drive to thinness	0.029 (0.100)	1.029	0.846–1.253
Bulimia	**0.768 (0.297)**	**2.155**	**1.204–3.858**
Body dissatisfaction	−0.159 (0.099)	0.853	0.702–1.036
Ineffectiveness	−0.004 (0.084)	0.996	0.844–1.175
Interpersonal distrust	−0.150 (0.111)	0.861	0.692–1.070
Interoceptive awareness	**0.360 (0.143)**	**1.433**	**1.083–1.070**
Maturity fears	0.071 (0.091)	1.074	0.898–1.284
Somatization	1.456 (0.875)	4.289	0.772–23.843
Obsessive–compulsive	**−2.372 (1.118)**	**0.093**	**0.010–0.834**
Interpersonal sensitivity	1.466 (0.930)	4.330	0.700–26.779
Depression	1.523 (1.222)	4.586	0.418–50.282
Anxiety	0.552 (0.791)	1.737	0.369–8.178
Hostility	−3.477 (1.299)	0.031	0.002–0.394
Phobic anxiety	−0.944 (0.996)	0.389	0.055–2.737
Psychoticism	−0.188 (1.136)	0.829	0.659–3.283
Sleep difficulties	0.386 (0.410)	1.471	0.659–3.283
Harm avoidance	0.033 (0.072)	1.034	0.897–1.191
Adults (R^2^ = 0.208)			
Drive to thinness	0.015 (0.053)	1.015	0.915–1.126
Bulimia	−0.022 (0.089)	0.978	0.822–1.165
Body dissatisfaction	0.033 (0.051)	1.033	0.935–1.143
Ineffectiveness	0.020 (0.062)	0.741	0.904–1.152
Interpersonal distrust	−0.160 (0.093)	0.852	0.711–1.022
Interoceptive awareness	0.003 (0.061)	1.003	0.890–1.130
Maturity fears	0.058 (0.068)	1.059	0.927–1.210
Somatization	0.245 (0.578)	1.277	0.412–3.962
Obsessive–compulsive	−0.899 (0.733)	0.411	0.098–1.729
Interpersonal sensitivity	−0.385 (0.565)	0.681	0.225–2.060
Depression	0.741 (0.864)	2.098	0.386–11.402
Anxiety	−0.134 (0.752)	0.875	0.201–3.816
Hostility	0.341 (0.515)	1.406	0.513–3.856
Phobic anxiety	1.079 (0.642)	2.943	0.835–10.365
Psychoticism	−0.090 (0.738)	0.914	0.215–3.881
Sleep difficulties	0.068 (0.268)	1.070	0.633–1.809
Harm avoidance	−0.012 (0.054)	0.989	0.889–1.100

Significant results are shown in bold. R^2^ was calculated using the Nagelkerke method.

## Data Availability

Data will be made available on request.
